# Molecular Characterizations of Double-Stranded RNA Degrading Nuclease Genes from *Ostrinia nubilalis*

**DOI:** 10.3390/insects11100652

**Published:** 2020-09-23

**Authors:** Anastasia M. W. Cooper, Huifang Song, Xuekai Shi, Zhitao Yu, Marcé Lorenzen, Kristopher Silver, Jianzhen Zhang, Kun Yan Zhu

**Affiliations:** 1 Department of Entomology, 123 Waters Hall, Kansas State University, Manhattan, KS 66506, USA; songhuifang88@126.com (H.S.); xuekaishi@163.com (X.S.); zhitaoy@ksu.edu (Z.Y.); ksilver@ksu.edu (K.S.); zjz@sxu.edu.cn (J.Z.); 2 Institute of Applied Biology, Shanxi University, Taiyuan 030006, China; 3 Department of Entomology and Plant Pathology, Campus Box 7613, North Carolina State University, Raleigh, NC 27695, USA; mdlorenz@ncsu.edu

**Keywords:** dsRNase, European corn borer, gene expression, REase, RNAi efficiency, substrate specificity

## Abstract

**Simple Summary:**

RNA interference is a gene suppression tool that uses double-stranded RNA to prevent specific genes from producing proteins. By targeting essential genes RNA interference can be developed for control of insect pests. Unfortunately, RNA interference is not equally effective for all insects. Previous investigation suggested that RNA is rapidly digested by unidentified components of body fluids in the European corn borer caterpillar. We characterized genes encoding proteins from European corn borer that are associated with RNA digestion in other insects. Our results suggest that two proteins (RNA interference efficiency-related nuclease and double-stranded RNA-degrading endonuclease 2) may be responsible for digesting RNAs in the European corn borer gut, whereas two other proteins (double-stranded RNA-degrading endonuclease 1 and double-stranded RNA-degrading endonuclease 4) may be responsible for digesting RNA in European corn borer body fluid. These findings suggest digestion of RNA in the European corn borer is likely due to the activity of these proteins. These findings provide information about the mechanism(s) influencing RNA stability in insects. The knowledge generated by this study will facilitate the development of strategies for enhancing RNA interference in insects.

**Abstract:**

Variable RNA interference (RNAi) efficiencies limit RNAi-based pest management strategies for many pests. Previous efforts to understand mechanisms contributing to low RNAi efficiency indicate that double-stranded RNA (dsRNA) is degraded in the European corn borer (ECB), *Ostrinia nubilalis,* due to nuclease activity. To investigate the contribution of dsRNA-degrading endonucleases (dsRNases) and lepidopteran-specific RNAi efficiency-related nucleases (REases) to dsRNA instability and low RNAi efficiency in ECB, five complementary DNAs putatively encoding four dsRNases (*OndsRNase1, 2, 3,* and *4*) and one REase (*OnREase*) were sequenced. Characterization of these transcripts revealed that substrate specificity might vary among the four dsRNases due to different amino acid combinations in the substrate-binding sites. Gene expression analysis indicated that *OndsRNase2* and *OnREase* were highly expressed in the larval gut, and *OndsRNase1* showed the highest expression in hemolymph, especially in older developmental stages. Transcript level analysis after dsRNA exposure revealed that expression of *OnREase* rapidly increased upon dsRNA ingestion or injection, whereas *OndsRNase4* expression only increased after long-term ingestion of dsRNA. While the biological function of these nucleases remains to be verified, our results suggest that OnREase and OndsRNase2, and OndsRNase1 and OndsRNase4 may be responsible for degradation of dsRNAs in the ECB gut and hemolymph, respectively, thereby contributing to low RNAi efficiency.

## 1. Introduction

RNA interference (RNAi)-mediated gene suppression uses double-stranded RNA (dsRNA) molecules to induce degradation of specific messenger RNAs before translation into proteins. RNAi pathways can be exploited by experimentally introducing dsRNA to silence specific genes, and elucidate their function or induce mortality [1]. As a result, RNAi is commonly used in the laboratory to analyze gene function, and RNAi-based pest management strategies are now being developed [2,3]. Unfortunately, RNAi is not equally effective in all insect taxonomic groups, limiting the ability of RNAi to control some insects, especially lepidopterans [4,5].

The European corn borer (ECB), *Ostrinia nubilalis* (Hübner), is one of the most destructive pests of corn in the USA and Europe [6]. *Bacillus thuringiensis* (Bt) Cry toxins and conventional insecticides are the most effective tools for controlling ECB, but the development of resistance in the field is compromising the effectiveness of traditional control methods [7,8], highlighting the need for developing new control strategies that utilize novel modes of action, such as RNAi. Unfortunately, ECB is one of the lepidopteran pests that exhibit very low RNAi efficiency [9,10], preventing the application of this promising new technology to the control of this notorious pest. Therefore, mechanistic research aimed at elucidating the factors limiting RNAi efficiency in ECB is needed to facilitate the development of RNAi-based pest management strategies in ECB and similar lepidopterans [5].

RNAi efficiency in insects can be limited by dsRNA instability, poor dsRNA internalization, lack of endosomal escape, deficient core RNAi machinery, and impaired systemic spreading of the silencing RNA molecules [11]. DsRNA-degrading endonucleases (as known as dsRNases) degrade dsRNA in various lepidopterans, orthopterans, and coleopterans reviewed in Cooper et al. [11]. DsRNases likely play important roles in limiting RNAi efficiency because they degrade dsRNA before dsRNA can be internalized into cells where the RNAi mechanism takes place [11]. In addition, lepidopteran-specific RNAi efficiency-related nucleases (REase) have been reported in *Ostrinia furnacalis* [12] and *Helicoverpa armigera* [13] and are alleged to contribute to the extremely low RNAi response of lepidopterans. Investigation of dsRNA stability in ECB demonstrated that dsRNA was rapidly degraded by enzymatic activity in larval ECB gut contents and hemolymph at physiologically relevant pH conditions [14]. However, the genes encoding nucleases that degrade dsRNA have not been identified or characterized in ECB and have been characterized only in a few insect species. Accordingly, we identified five complementary DNAs (cDNAs) putatively encoding four dsRNases and one REase from ECB. In addition, we characterized gene expression profiles from ECB to determine the likelihood of these nucleases contributing to the instability of dsRNA in hemolymph and gut contents from ECB. The results support the hypothesis that dsRNA-degrading nucleases contribute to low dsRNA stability in ECB, and thus low RNAi efficiency in ECB.

## 2. Materials and Methods

### 2.1. Insect Rearing

The ECB used in this study originated from French Agricultural Research (Lumberton, MN, USA), and were continuously reared in the laboratory at Kansas State University (Manhattan, KS, USA) as described in Cooper et al. [14]. 

### 2.2. Sequencing of dsRNase and REase cDNAs

To amplify cDNA derived from putative ECB dsRNase and REase transcripts, specific primers were designed based on transcriptome data from *O. furnacalis*. Primer pairs (Table S1) were designed manually or using the National Center for Biotehnology Information’s (NCBI’s) Primer-BLAST web tool (https://www.ncbi.nlm.nih.gov/tools/primer-blast/) and then ordered from Invitrogen (Carlsbad, CA, USA). Total RNA was extracted using TRIzol reagent (Invitrogen, Carlsbad, CA, USA) following the manufacturer’s instructions and resuspended in DEPC-treated water. The quality and quantity of total RNA were assessed using a NanoPhotometer (Implen, Westlake Village, CA, USA). One microgram of total RNA from each sample was then treated with DNase I (Thermo Fisher Scientific, Waltham, MA, USA) to remove genomic DNA and converted to cDNA using the Prime-Script RT Reagent Kit (Takara, Mountain View, CA, USA) using both random hexamers and oligo(dT) primers supplied in the kit. Overlapping PCR products of the expected sizes were amplified from cDNA obtained from pooled last-instar larvae. Complementary DNA fragments of the genes were amplified by Advantage 2 polymerase (Takara, Mountain View, CA, USA). PCR products were separated by electrophoresis on 1% agarose gels. Target bands were purified using the Gel/PCR DNA fragments extraction kit (IBI Scientific, Road Dubuque, IA, USA) and directly ligated into the pCR-II vector (Invitogen, Carlsbad, CA, USA) for transformation into DH5α competent *Escherichia coli* cells using heat shock. Positive plasmids containing the target gene fragment were identified by colony PCR using universal M13 primers. Purified plasmids were sent to Genewiz LLC (South Plainfield, NJ, USA) for Sanger sequencing. Overlapping sequenced fragments spanning the length of the transcripts were aligned with Sequencher 5.0 DNA Sequence Analysis software (Gene Codes Corporation, Ann Arbor, MI, USA) and used to design specific primers for 5′- and 3′- rapid amplification of cDNA ends (RACE) using a Smarter RACE Amplification kit (Takara, Mountain View, CA, USA) according to the manufacturer’s protocol. Finally, specific primers designed near the start and stop codon were used to amplify the entire open reading frame (ORF) and confirm the full-length sequences (Table S1). Putative nucleotide sequences were translated using Gene Runner ver. 3.01 software (Hasting Software Inc., Las Vegas, NV, USA), and finally submitted to GenBank. The molecular mass and isoelectric points were predicted with the isoelectric.org web tool. 

### 2.3. Phylogenetic, Domain, and Peptide Analyses of Predicted Proteins

To verify the identity of the putative dsRNA-degrading nucleases, multiple sequence alignments, phylogenetic analysis, domain comparisons, and subcellular localization predictions were performed. Amino acid residues in the active site were also compared to investigate possible variation in substrate specificity among OndsRNase proteins. Amino acid sequences were obtained from NCBI, the National Agricultural Library (NAL) i5k Workspace, the supplementary information from Swevers et al. [15] and Guan et al. [12], or predicted based on cDNAs sequenced by our group at either Kansas State University in the USA or at Shanxi University in China as described above. Amino acid sequences for REases were obtained by pBLAST of OnREase against the NCBI database for each insect order.

Multiple sequence alignments were performed with the MUSCLE method using MEGA7 software [16] and visualized with GeneDoc 2.7 (https://genedoc.software.informer.com/). Phylogenetic analyses were also constructed using MEGA7 following the Maximum Likelihood procedure described by Hall [17]. Bootstrap consensus trees inferred from 500 replicates are shown, with branches corresponding to partitions reproduced in less than 50% bootstrap replicates collapsed. Full-length amino acid sequences were used for the REase tree, whereas only the endonuclease domain and surrounding 20 amino acids were used for the dsRNase tree to achieve better resolution among clades. 

Protein domains were predicted with the Pfam 32.0 web tool (http://pfam.xfam.org/search/sequence) [18]. Signal peptides were predicted with the SignalP-5.0 Server web tool (http://www.cbs.dtu.dk/services/SignalP/) [19], and the Target P-2.0 Server web tool. (http://www.cbs.dtu.dk/services/TargetP/) [20]. Subcellular localization was predicted with the WoLF PSORT computational web tool (https://www.genscript.com/wolf-psort.html) [21], the Euk-mPLoc 2.0 server (http://www.csbio.sjtu.edu.cn/bioinf/euk-multi-2/) [22], and the iLoc-Animal server (http://jci-bioinfo.cn/iLoc-Animal) [23].

### 2.4. Stage- and Tissue-Specific Expression Profiles

To examine where and when transcripts encoding putative nucleases were expressed in ECB, tissue-specific and developmental stage-specific expression profiles were generated for each nuclease gene. Whole-body and tissue samples were collected from laboratory-reared ECB in TRIzol reagent (Invitrogen, Carlsbad, CA, USA) for the extraction of total RNA. At least three individuals were used per replicate, and three biological replicates per treatment. The homogenized samples were frozen at −80 °C until further processing. 

Total RNA was extracted following the manufacturer’s instructions and resuspended in DEPC-treated water. The quality and quantity of total RNA were assessed using a NanoPhotometer (Implen, Westlake Village, CA, USA). One microgram of total RNA from each sample was treated with DNase I (Thermo Fisher Scientific, Waltham, MA, USA) to remove genomic DNA, and then converted to cDNA using the EasyScript cDNA synthesis kit (Applied Biological Materials, Richmond, BC, Canada) following the manufacturer’s instructions. Obtained cDNA was diluted 5-fold with nuclease-free water for use as template for expression analysis using reverse transcription quantitative PCR (RT-qPCR). 

### 2.5. RT-qPCR

RT-qPCR was used to examine tissue and developmental stage-specific expression of nuclease genes in ECB. RT-qPCR was performed in accordance with the minimum information for publication of quantitative real-time PCR experiment (MIQE) guidelines [24] as described in Cooper et al. [14]. 

To provide better visualization of fluctuations in gene expression over the time points analyzed, relative expression was calculated as the expression of the target gene relative to the geometric mean of the reference genes (i.e., ΔCt rather than ΔΔCt [25]). First, the mean Ct values of all technical replicates were normalized to the geometric mean of ribosomal protein S3 gene (*Rps3*, DQ988989) and elongation factor-1 alpha gene (*Ef1a*, AF173392) to calculate ΔCt. Finally, fold change for each biological replicate was calculated, subjected to statistical analysis, and the mean and standard error of each treatment graphed. Ct values over 32 were considered nondetectible, and a fold change of zero was used for analysis. NormFinder [26], geNorm [27], BestKeper [28], and RefFinder (http://150.216.56.64/referencegene.php) were used to verify the stability of reference genes across ECB tissues and developmental stages. Percent suppression of the target gene was calculated as [(control-target)/control) × 100%]. 

### 2.6. Synthesis of dsRNAs

Long dsRNAs (500 bp) that target either an endogenous gene encoding lethal giant larvae protein (OnLgl; MT467568) from the ECB, or an exogenous gene encoding enhanced green fluorescent protein (GFP; LC336974.1), were synthesized for ingestion and injection experiments in ECB larvae to assess the transcriptional responses of nuclease genes to dsRNA treatment. DsRNA was synthesized and purified as described in Cooper et al. [14]. 

### 2.7. Transcriptional Responses of Nuclease Genes to dsRNA Exposure

To investigate short- and long-term transcriptional responses of nuclease genes from ECB to dsRNA exposure, three individuals were collected at various time points after ingestion or injection of dsRNA into second-instar larvae for expression analysis with RT-qPCR. Second instar larvae were selected for this investigation because the dsRNA feeding assay was optimized for young larvae and microinjection is less harmful to second instars than neonate larvae. Experiments were designed in a two-way factorial treatment structure so that significant effects on relative gene expression, weight, and survivorship due to dsRNA (e.g., ds*OnLgl*, ds*GFP*, water), nuclease inhibitor (e.g., 0, 10, 20 mM Zn^2+^), or interaction between both factors could be investigated. 

Injection of dsRNA was performed using a Nanoinject II system (Drummond Scientific, PA, USA) coupled with a SYS-Micro4 controller (World Precision Instruments, Sarasota, FL, USA). Each 125-nL injection contained 500 ng of dsRNA with and without reagent delivered at the intersegmental membrane of abdominal segments A5–A6. An equal volume of nuclease-free water or phosphate buffered saline (PBS; pH 7.0) was used for control injections. In total, 20–25 individuals were injected per replicate and placed on artificial diet inside of 37-mL clear plastic cups sealed with oversnap caps (Frontier Agricultural Sciences, Newark, DE, USA). Larvae were maintained under rearing conditions until sampling. 

Ingestion of dsRNA was performed based on Khajuria et al. [9,10]. Ten micrograms of dsRNA with and without each reagent were applied to artificial diet. An equal volume of nuclease-free water or PBS (pH 8.0) was used for ds*GFP* controls. Nitrogen gas was used to dry dsRNA/reagent solutions (10 µg/larvae) onto 20 mg squares of artificial diet inside individual Bio-Assay Tray cells (Frontier Agricultural Sciences, Newark, DE, USA). Then, 25 larvae were then transferred into each prepared cell using a fine-point paintbrush and sealed inside with Bio-Assay Tray Lids (Frontier Agricultural Sciences, Newark, DE, USA). Larvae were then allowed to feed under rearing conditions for 3 days. On the third day (once all the diet had been consumed), the larvae were transferred to a new cell containing a dsRNA/reagent-treated 40 mg diet square, and then allowed to feed for another 3 days (until all diet was again consumed). Thus, an estimated 20 µg of dsRNA was fed to each larva over a period of 6 days. Larvae were maintained under rearing conditions until sampling.

Sampling for expression analysis was performed at 3, 6, 12, and 24 h after the start of dsRNA exposure. Three individuals (the largest, the smallest, and an intermediate-sized individual) from each replicate of each treatment were pooled at each time point and homogenized in TRIzol reagent (Invitrogen, Carlsbad, CA, USA) and frozen at −80 °C until further processing for RT-qPCR. The effects on transcript levels were calculated as described above.

### 2.8. Statistical Analyses

Statistical differences between treatment means were assessed in Minitab 18 with either a one- or two-way ANOVA followed by a Tukey HSD test. Significance levels (α) were set at 0.05 for the entire family of comparisons and significant p-values indicated with an asterisk. All data sets were subjected to the Anderson–Darling normality test and Levene’s test for equal variance to verify statistical assumptions were met. Data that did not meet the assumptions were subjected to either a Kruskal–Wallis one-way ANOVA on ranks test, or to dual Friedman’s two-way analysis by ranks tests followed by multiple Wilcoxon signed-rank tests for each desired pairwise comparison. The Bonferroni correction was used to control for type I error occurring from the use of multiple parametric statistical tests. In all experiments, treatments were replicated at least three times. 

## 3. Results

### 3.1. Sequencing and Characterization 

**Figure 1:**
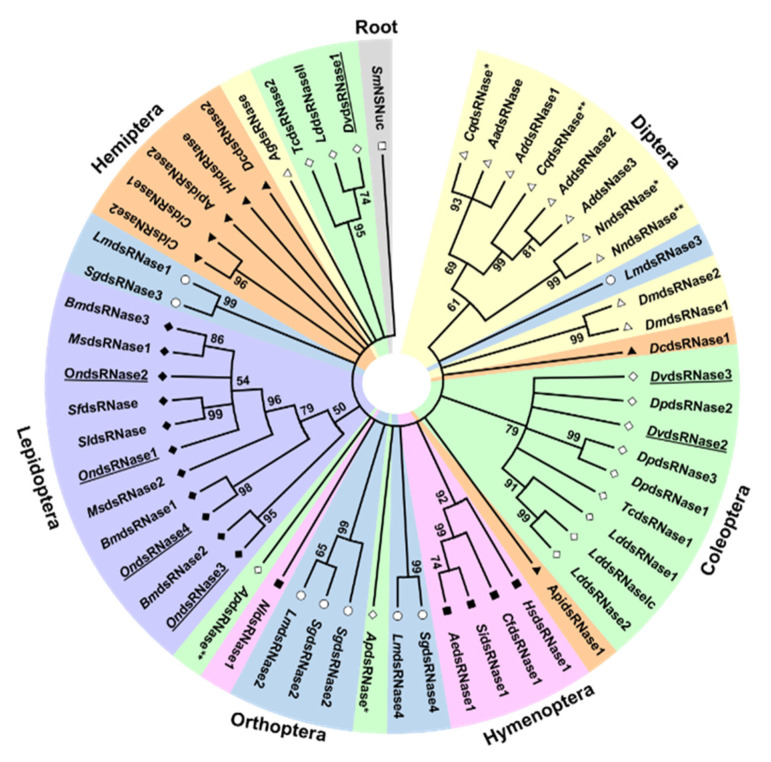
Phylogenetic tree showing the relationships between insect dsRNase proteins and bacterial nonspecific nuclease. Bootstrap support is indicated at internal nodes. Different shapes and shading denote different insect orders. The species and gene accession numbers corresponding to each sequence label are as follows for each order. Asterisks (*, **) differentiate unnumbered dsRNases from the same species. Diptera: AadsRNase, *Aedes aegypti* (EAT42072); CqdsRNase*, *Culex quinquefasciatus* (EDS34867.1); AddsRNase1, *Anopheles darling* (ETN62076.1); CqdsRNase**, *C. quinquefasciatus* (EDS38458.1); AddsRNase2, *A. darling* (ETN61460.1); AddsRNase3, *A. darling* (ETN61459.1); NndsRNase*, *Nyssomyia neivai* (JAV11177.1); NndsRNase**, *N. neivai* (JAV11176.1); DmdsRNase1, *Drosophila melanogaster* (AAF49206.1). DmdsRNase2, *D. melanogaster* (AAF49208.1); AgdsRNase, *Anopheles gambiae* (XP_320813.4). Coleoptera: DvdsRNase2, *Diabrotica virgifera virgifera* (MT653319); DvdsRNase3, *D. v. virgifera* (MT653320); DpdsRNase2, *Dendroctonus ponderosae* (ERL84039.1); TcdsRNase1, *Tribolium castaneum* (XP_970494.1); DpdsRNase3, *D. ponderosae* (AEE63490.1); DpdsRNase1, *D. ponderosae* (ENN82866.1); LddsRNase1/Ib*, Leptinotarsa decemlineata* (APF31792.1); LddsRNase2/Ia*, L. decemlineata* (APF31793.1); LdsRNaseIc*, L. decemlineata* (Swevers et al., 2013); ApdsRNase*, *Agrilus planipennis* (XP_018334885.1); ApdsRNase**, *A. planipennis* (XP_018323185.1); TcdsRNase2, *T. castaneum* (XP_015840884.1); DvdsRNase1, *D. v. virgifera* (MT653318); LddsRNaseII*, L. decemlineata* (Swevers et al., 2013). Hymenoptera: NldsRNase1, *Neodiprion lecontei* (XP_015515106.1); AedsRNase1, *Acromyrmex echinatior* (XP_011064189.1); SidsRNase1, *Solenopsis invicta* (XP_011156845.1); CfdsRNase1, *Camponotus floridanus* (XP_011263277.1); HsdsRNase1, *Harpegnathos saltator* (XP_011148137.2). Orthoptera: LmdsRNase3, *Locusta migratoria* (KY386893); SgdsRNase2, *Schistocerca gregaria* (AHN55089.1/APF31794.1); LmdsRNase2, *L. migratoria* (ARW74135.1); SgdsRNase1, *S. gregaria* (AHN55088.1); SgdsRNase4, *S. gregaria* (AHN55091.1); LmdsRNase4, *L. migratoria* (KY386894); SgdsRNase3, *S. gregaria* (AHN55090.1); LmdsRNase1, *L. migratoria* (ARW74134.1). Lepidoptera: BmdsRNase2, *Bombyx mori* (NP_001091744.1); OndsRNase1, *Ostrinia nubilalis* (MT524713); OndsRNase4, *O. nubilalis* (MT524714); BmdsRNase1, *B. mori* (XP_004922835.1); OndsRNase*1*, *O. nubilalis* (MT524715); OndsRNase2, *O. nubilalis* (MT524712); SldsRNase, *Spodoptera litura* (CAR92522.1); SfdsRNase, *Spodoptera frugiperda* (CAR92521.1); MsdsRNase2, *M. sexta* (Msex2.04564); *Bm*dsRNase3/AlkNuc, *B. mori* (BAF33251.1); MsdsRNase1, *M. sexta* (Msex2.04563). Hemiptera: DcdsRNase1, *Diaphorina citri* (XP_017297751.1); ApidsRNase1, *A. pisum* (XP_003242652.1); CldsRNase1, *Cimex lectularius* (XP_014241898.1); CldsRNase2, *C. lectularius* (XP_014241376.1); HhdsRNase*, Halyomorpha halys* (XP_014282547.1); DcdsRNase2, *D. citri* (XP_008483858.1); ApidsRNase2, *Acyrthosiphon pisum* (XP_003248225.1). Root: Enterobacteriales: SmNSNuc, *Serratia marcescens* (AAA26560.1).

**Figure 2:**
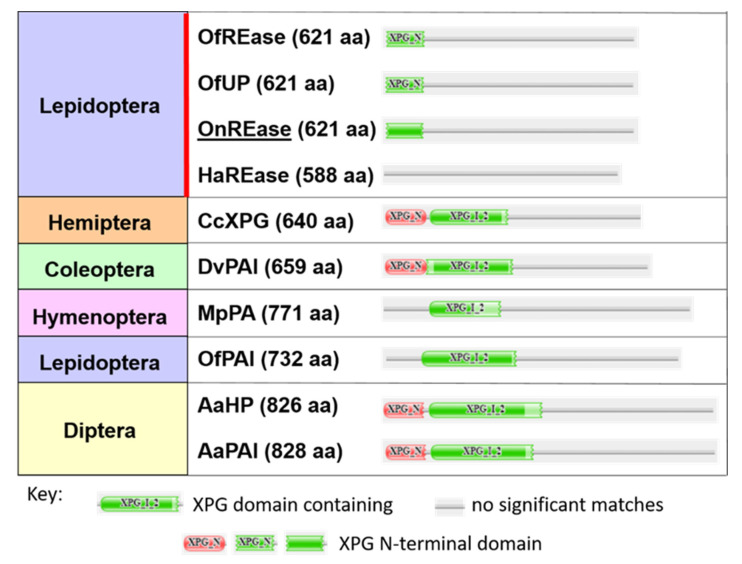
Comparison of domain architecture showing the differences between lepidopteran REase (red line) and insect protein asteroids. The species and gene accession numbers corresponding to each sequence label are as follows for each order. Lepidoptera: OfREase, *Ostrinia furnacalis* (XP_028162616.1); OfUP, *O. furnacalis* (XP_028162616.1); OnREase, *Ostrinia nubialis* (MT524716); HaREase, *Helicoverpa armigera* (XP_021192733.1); OfPAI, *O. furnacalis* (XP_028160864.1). Hemiptera: CcXPG, *Cinara cedri* (VVC40419.1)/Coleoptera: DvPAl, *Diabrotica virgifera virgifera* (XP_028153404.1). Hymentoptera: MpPA, *Monomorium pharaonis* (XP_012539364.1). Diptera: AaHP, *Aedes albopictus* (KXJ83147.1); AaPAl, *A. albopictus* (XP_019538095.2); Abbreviations: RNAi-efficiency related nuclease, REase; uncharacterized protein, UP; hypothetical protein, HP; XPG N-terminal PIN domain-like endonuclease, XPG; protein asteroid, PA; protein asteroid-like, PAl.

**Figure 3:**
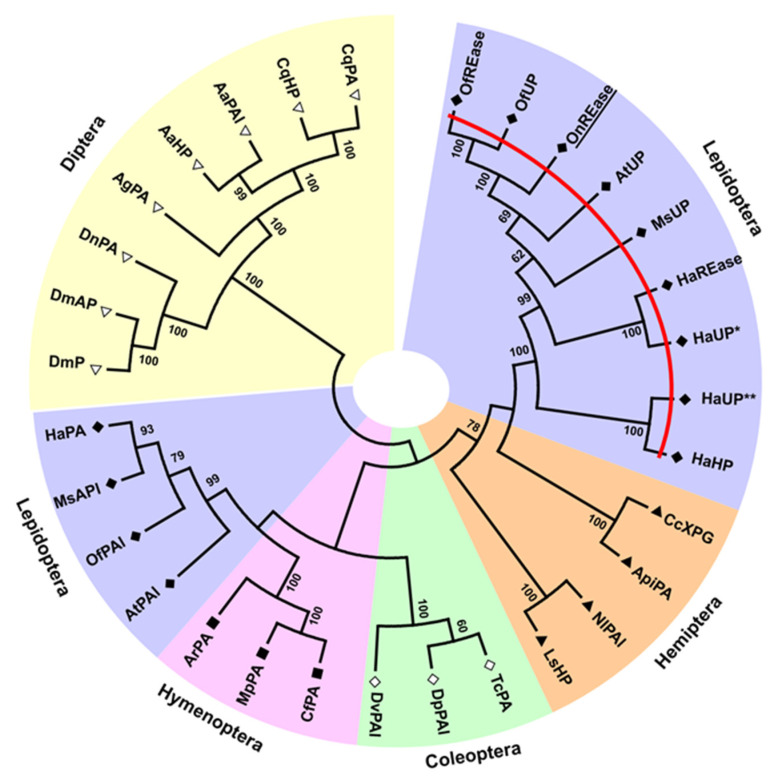
Phylogenetic tree showing the relationships between REase proteins (red line), protein asteroids, and unknown/hypothetical proteins from insects. Bootstrap support is indicated at internal nodes. Different shapes and shading denote different insect orders. The species and gene accession number corresponding to each sequence label is as follows for each order. Asterisks (*,**) differentiate unnumbered proteins from the same species. Lepidoptera: OfREase, *Ostrinia furnacalis* (XP_028162616.1); OfUP, *O. furnacalis* (XP_028162616.1); OnREase, *Ostrinia nubialis* (MT524716); AtUP, *Amyelois transitella* (XP_013194003.1); MsUP, *Manduca sexta* (XP_030022308.1); HaREase, *Helicoverpa armigera* (XP_021192733.1); HaUP*, *H. armigera* (XP_021192733.1); HaUP**, *H. armigera* (XP_021195627.1); HaHP, *H. armigera* (PZC74001.1); AtPAI, *A. transitella* (XP_013200440.1); OfPAI, *O. furnacalis* (XP_028160864.1), MsPAI*, M. sexta* (XP_030035285.1); HaPA, *H. armigera* (XP_021187181.1). Hemiptera: CcXPG, *Cinara cedri* (VVC40419.1); ApiPA, *Acyrthosiphon pisum* (XP_008180179.1); NlPAl, *Nilaparvata lugens* (XP_022192084.1); LsHP, *Laodelphax striatellus* (RZF34787.1). Coleoptera: TcPA*, Tribolium castaneum* (XP_975212.1); DpPAl, *Dendroctonus ponderosae* (XP_019754476.1); DvPAl, *Diabrotica virgifera virgifera* (XP_028153404.1). Hymentoptera: CfPA, *Camponotus floridanus* (XP_011264114.1); MpPA, *Monomorium pharaonis* (XP_012539364.1); ArPA, *Athalia rosae* (XP_012251909.1). Diptera: DmP, *Drosophila melanogaster* (AAK93494.1); DmAP, *D. melanogaster* (NP_523451.2); DnPA, *Drosophila navojoa* (XP_030240503.1); AgPA, *Anopheles gambiae* (XP_318635.3); AaHP, *Aedes albopictus* (KXJ83147.1); AaPAl, *A. albopictus* (XP_019538095.2); CqHP, *Culex quinquefasciatus* (XP_001870537.1); CqPA, *C. quinquefasciatus* (XP_001846107.1). No blast hits were found for Orthoptera. Abbreviations: RNAi-efficiency related nuclease, REase; uncharacterized protein, UP; hypothetical protein, HP; XPG N-terminal PIN domain-like endonuclease, XPG; protein asteroid, PA; protein asteroid-like, PAl.

Our sequence analysis revealed four dsRNase transcripts and a single REase transcript expressed in ECB (Table 1). All four predicted OndsRNase proteins were similar in length (Table 1), contained an extracellular secretion peptide (Table 1, Figure S1) and single DNA/RNA nonspecific endonuclease (IPR001604) domain (Figure S1), and clustered in the main lepidopteran clade on a phylogenetic tree of insect dsRNase proteins (Figure 1). Conversely, OnREase was predicted to be intracellular (Table 1) and included an XPG N-terminal domain (PF00752) (Figure 2) as well as three conserved residues common to PIN-family domains (Figure S2). Phylogenetic analysis indicated that OnREase clustered with other REase and uncharacterized/hypothetical proteins from lepidopterans, but separately from protein asteroid proteins, protein asteroid-like proteins, and uncharacterized/hypothetical proteins from other insects (Figure 3). Comparison of domain architecture indicated that most proteins from the REase cluster are predicted to have an XPG N-terminal domain (PF00752), whereas all proteins from the asteroid cluster are predicted to have an XPG domain-containing (PF12813) region (Figure 2). The XPG N-terminal domain was not identified in HaREase, but was identified in OnREase and OfREase.

**Figure 4:**
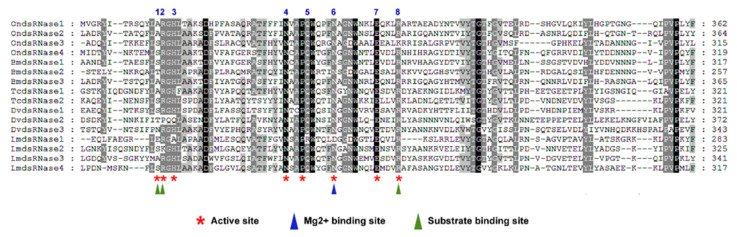
Multiple sequence alignments showing conserved residues in the active sites of insect dsRNases and a bacterial nonspecific nuclease. The eight amino acid residues that form the active site are indicated by a red asterisk and numbered along the top. Amino acid residues that participate in the substrate-binding site and Mg^2+^ binding site are indicated with green and blue triangles, respectively. Positions with 100% conservation are indicated by an asterisk. The species and gene accession number corresponding to each sequence label is as follows: SmNSNuc, *Serratia marcescens* (AAA26560.1); BmdsRNase1, *B. mori* (XP_004922835.1); BmdsRNase2, *Bombyx mori* (NP_001091744.1); BmdsRNase3/AlkNuc, *B. mori* (BAF33251.1); OndsRNase2, *Ostrinia nubilalis* (MT524712); OndsRNase3, *O. nubilalis* (MT524713); OndsRNase4, *O. nubilalis* (MT524714); OndsRNase1, *O. nubilalis* (MT524715); DvdsRNase1, *Diabrotica virgifera virgifera* (MT653318); DvdsRNase2, *D. v. virgifera* (MT653319); DvdsRNase3, *D. v. virgifera* (MT653320); TcdsRNase1, *Tribolium castaneum* (XP_970494.1); TcdsRNase2, *T. castaneum* (XP_015840884.1); LmdsRNase1, *L. migratoria* (ARW74134.1); LmdsRNase2, *L. migratoria* (ARW74135.1); LmdsRNase3, *Locusta migratoria* (KY386893); LmdsRNase4, *L. migratoria* (KY386894).

Multiple sequence alignments of predicted dsRNase proteins revealed that the first and eighth key residues of the predicted dsRNase active site were variable among dsRNases from ECB as well as from other insects (Figure 4). OndsRNase1 and OndsRNase2 both have an alanine (A) and an arginine (R) at positions 1 and 2 in the active site. Conversely, OndsRNase3 and OndsRNase4 both have a serine (S) and arginine (R) at positions 1 and 2 in the active site.

**Figure 5:**
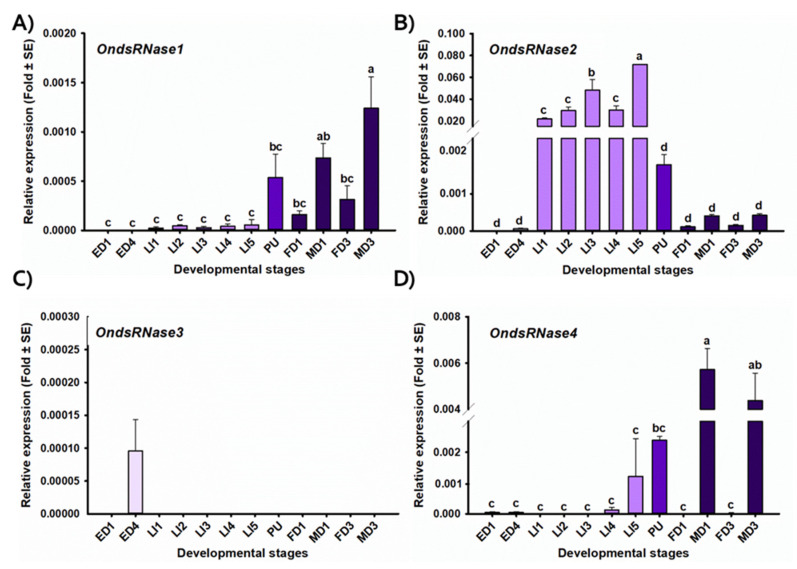
Expression profiles of OndsRNase transcripts in different developmental stages. Relative expression level (fold plus or minus standard error) of (**A**) *OndsRNase1,* (**B**) *OndsRNase2,* (**C**) *OndsRNase3*, and (**D**) *OndsRNase4* in 1-day-old eggs (ED1), 4-day-old eggs (ED4), first-instar larvae (LI1), second-instar larvae (LI2), third-instar larvae (LI3), fourth-instar larvae (LI4), fifth-instar larvae (LI5), pupae (PU), 1-day-old adult females (FD1), 1-day-old adult males (MD1), 3-day-old adult females (FD3), and 3-day-old adult males (MD3). Fold change is relative to the expression of reference genes only (ΔCt). Significant differences among the different developmental stages are indicated by different letters on the bars of the standard errors.

Assessment of developmental stage-specific expression profiles (Figure 5) indicated that *OndsRNase1* expression was lowest in eggs and larvae but increased throughout the rest of the developmental cycle, peaking in adult males [F(11,24) = 8.76, *p* < 0.0001 *, α<0.05]. *OndsRNase2* was detectable in all ECB developmental stages except 1-day-old eggs, but expression was highest in larval stages [F(11,23) = 45.83, *p* < 0.0001 *]. *OndsRNase3* expression was only detectable in 4-day-old eggs. *OndsRNase4* was highly expressed in pupae and adult males, and expression of OndsRNase4 was highest in adult males [F(11,24) = 12.49, *p* < 0.0001 *]. 

**Figure 6:**
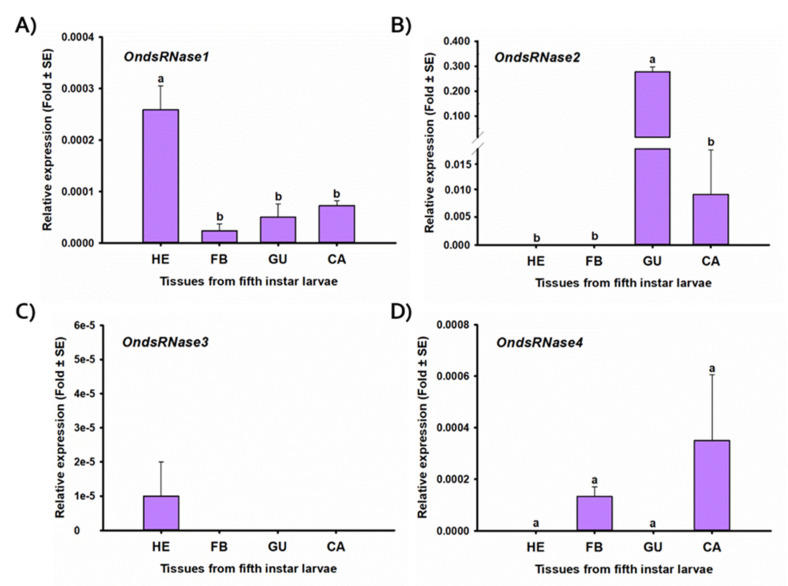
Expression profiles of OndsRNase transcripts in four different tissues from fifth-instar larvae. Relative expression level (fold plus or minus standard error) of (**A**) *OndsRNase1,* (**B**) *OndsRNase2,* (**C**) *OndsRNase3*, and (**D**) *OndsRNase4* in hemolymph (HE), fat body (FB); gut (GU), and carcass (CA). Fold change is relative to the expression of reference genes only (ΔCt). Significant differences among different tissues are indicated by different letters on the bars of the standard errors.

Tissue-specific expression profiles from fifth-instar larvae (Figure 6) indicated that *OndsRNase1* was detectable in all tissues investigated, with the highest expression in hemolymph [F(3,8) = 15.09, *p* = 0.001 *]. *OndsRNase2* was highly expressed in the gut and to a much lesser extent in the carcass, but not detectable in hemolymph or fat bodies [F(3,8) = 209.26, *p* < 0.0001 *]. *OndsRNase3* expression was only detectable in the hemolymph. *OndsRNase4* was expressed in carcass and fat bodies, but not detectable in gut and hemolymph [F(3,8) = 1.62, *p* = 0.260]. 

**Figure 7:**
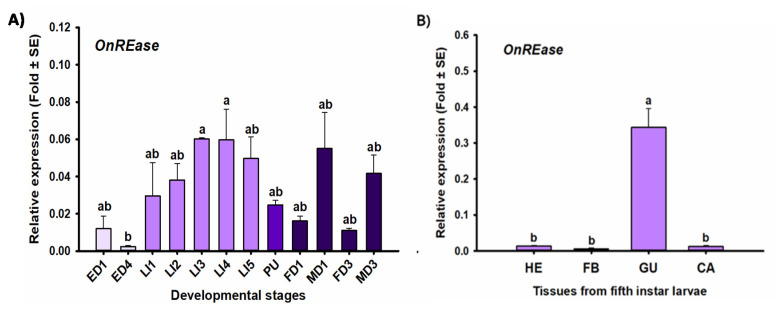
Expression profiles of *OnREase* in different developmental stages (**A**) and tissues from fifth-instar larvae (**B**). Relative expression level (fold plus or minus standard error) of *OnREase* in 1-day-old eggs (ED1) 4-day-old eggs (ED4), first-instar larvae (LI1), second-instar larvae (LI2), third-instar larvae (LI3), fourth-instar larvae (LI4), fifth-instar larvae (LI5), pupae (PU), 1-day-old adult females (FD1), 1-day-old adult males (MD1), 3-day-old adult females (FD3), and 3-day-old adult males (MD3)*,* as well as in hemolymph (HE), fat bodies (FB); gut (GU), and carcass (CA). Fold change is relative to the expression of reference genes only (ΔCt). Significant differences among treatments are indicated by different letters.

*OnREase* was detected in all developmental stages and tissues investigated (Figure 7). Developmental stage-specific expression of *OnREase* was highest in third- and fourth-instar larvae and lowest in 4-day-old eggs, though these were not significantly different from the other developmental stages examined [F(11,23) = 3.74, *p* = 0.004 *]. Tissue-specific expression of *OnREase* was highest in the gut of fifth-instar larvae, compared to other tissues investigated [F(3,8) = 40.85, *p* < 0.0001 *]. 

### 3.2. Transcriptional Responses to dsRNA 

**Figure 8:**
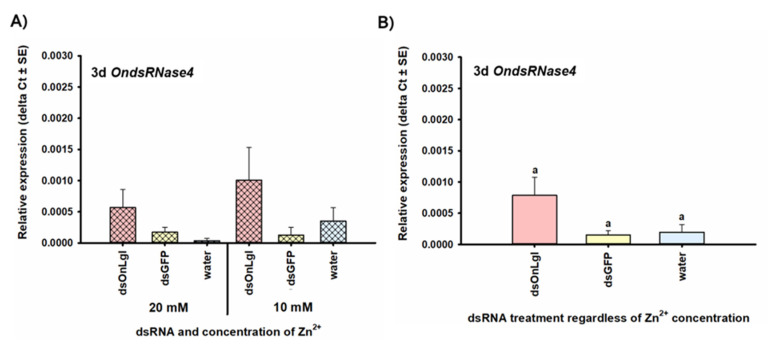
Relative expression of *OndsRNase4* after 3 days of feeding on dsRNA/nuclease inhibitor for all treatment groups (**A**), and for main effects due to dsRNA treatment (**B**). Relative expression of *OndsRNase4* in second-instar larvae, 3 days after the start of feeding on artificial diet treated with various combinations of dsRNA and nuclease inhibitor (Zn^2+^). Expression levels are relative to reference genes only (ΔCt).

**Figure 9:**
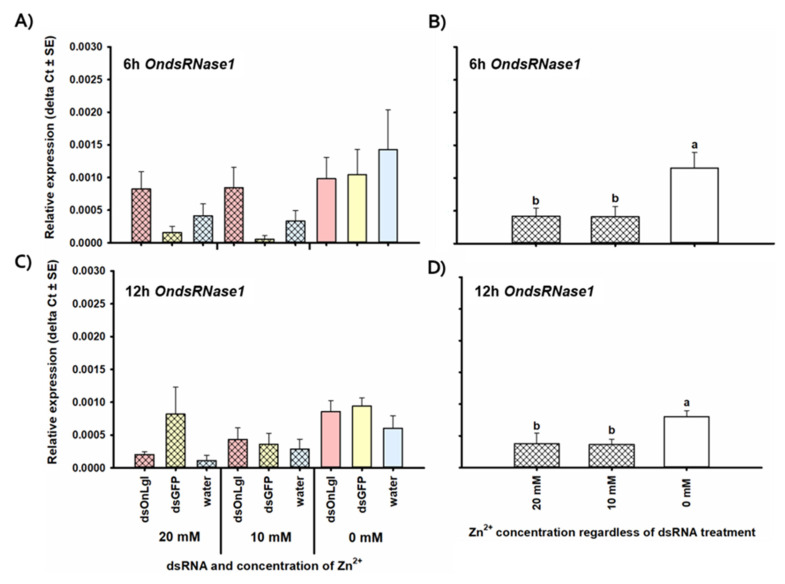
Relative expression of *OndsRNase1* 6 (**A**,**B**) and 12 h (**C**,**D**) after injection of dsRNA/nuclease inhibitor for all treatment groups (**A**,**C**) and for main effects due to Zn^2+^ concentration (**B**,**D**). Relative expression of *OndsRNase1* 6 and 12 h after injection of various combinations of dsRNA and Zn^2+^ into second-instar larvae. Expression levels are relative to reference genes only (ΔCt). Significant differences among treatments are indicated by different letters.

None of the OndsRNase genes were significantly upregulated after dsRNA ingestion or injection at any of the short-term time points investigated. Transcript levels of OndsRNase2 and OndsRNase3 were unaffected by dsRNA/Zn^2+^ injection and ingestion at all of the time points investigated (data not shown). In addition, the expression of *OndsRNase4* was unaffected by dsRNA/Zn^2+^ injection (data not shown). However, 3 days after the start of dsRNA feeding, expression of *OndsRNase4* increased by 5.3-fold in the ds*OnLgl* fed treatment group compared to the ds*GFP* fed treatment group [F(2, 12) = 3.50, *p* = 0.064] (Figure 8). Conversely, *OndsRNase1* expression was unaffected by dsRNA/Zn^2+^ feeding (data not shown), but decreased by 2.8-fold on average 6 h after Zn^2+^ injection [F(2, 17) = 5.24, *p* = 0.017 *] (Figure 9A,B), and by 2.1-fold 12 h after Zn^2+^ injection [F(2, 18) = 5.03, *p* = 0.018 *] (Figure 9C,D). 

**Figure 10:**
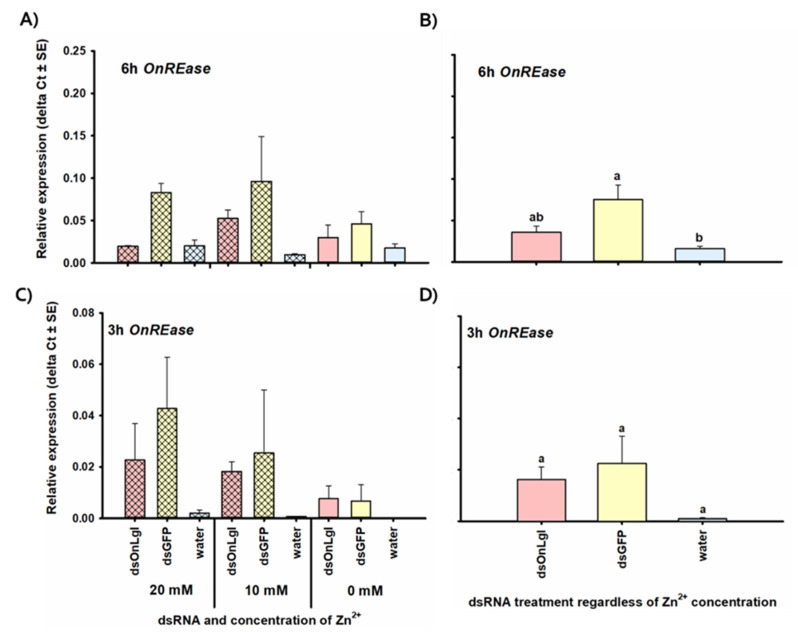
Relative expression of *OnREase* 6 h after injection (**A**,**B**) and 3 h after feeding (**C**,**D**) of dsRNA/inhibitor for all treatment groups (**A**,**C**) and for main effects due to dsRNA treatment (**B**,**D**). Mean fold change in the relative expression of *OndsREase* 6 h after injection and 3 h after feeding of various combinations of dsRNA and Zn^2+^ into second-instar larvae. Fold change is relative to the expression of reference genes only. Significant differences among treatments are indicated by different letters (ΔCt).

Transcript levels of *OnREase* were significantly upregulated after both dsRNA ingestion and injection. *OnREase* expression significantly increased by 4.7-fold 6 h after injection of ds*GFP* compared to water injection, regardless of Zn^2+^ concentration [F(2,17) = 6.67, *p* = 0.007 *]; however, there was no significant difference between *OnREase* expression in the ds*OnLgl* injected treatment group versus the water-only treatment group at 6 h after injection (Figure 10B). Transcript levels of *OnREase* were also upregulated 22.0-fold 3 h after the start of dsRNA feeding, regardless of Zn^2+^ concentration [F(2,18) = 3.10, *p* = 0.070] (Figure 10C,D), but not at any of the other time points investigated.

## 4. Discussion

Previously, we showed that dsRNA was highly unstable in ECB gut contents and hemolymph under physiological conditions, and the degradation of dsRNA is likely enzymatic in nature [14]. In the present study, we identified and characterized four dsRNase transcripts and one REase transcript in ECB that are likely associated with those previous observations. In other insects, dsRNA-degrading nucleases, such as dsRNases and REases, have been implicated in limiting RNAi efficiency by degrading dsRNA in *Bombyx mori* [29,30,31], *Schistocerca gregaria* [32], *Leptinotarsa decemlineata* [33], *Locusta migratoria* [34,35], *O. furnacalis* [12], *H. armigera* [13], and *Aedes aegypti* [36]. Most lepidopteran insects appear to contain three to four dsRNase transcripts (Figure 1) and one to three REase transcripts (Figure 3). Insects in other orders, lack REases, and contain only one to three dsRNases, supporting the hypothesis that differences in dsRNA-degrading nuclease activity may contribute to the low RNAi efficiency exhibited by many lepidopterans, including ECB. 

Comparisons of domain architectures for predicted REase proteins indicated that OnREase contains an XPG N-terminal domain (a single stranded, structure-specific DNA endonuclease domain) belonging to the PIN-domain nuclease family, similar to OfREase and OfUP from *O. furnacalis* (Figure 2). However, no significant domain matches were identified in HaREase from *H. armigera* (Figure 2), despite the presence of the three conserved residues at the N-terminus, which are common to all PIN-domain family members (Figure S2). Perhaps these differences in domain architecture can help explain why RNAi-mediated suppression of OfREase resulted in enhanced RNAi efficiency in *O. furnacalis* [12], but knockout of HaREase with CRISPR-Cas9 did not improve RNAi efficiency in *H. armigera* [13]. Similarities in domain structure between OnREase and OfREase (Figure 2), their close phylogenetic relationship (Figure 4), and high expression in the gut (Figure 8B) suggest that OnREase may play a similar role in dsRNA degradation as OfREase [12]. In addition, subcellular localization predictions and signal peptide predictions indicate that OnREase is an intracellular nuclease located in the cytoplasm. If so, OnREase and other REases likely degrade dsRNA after it is internalized into cells.

Unlike REases, all insect dsRNases were predicted to have similar domain structures and contain a signal peptide for extracellular secretion (Table 1), as previously described for insect dsRNases [37]. Interestingly, a comparison of conserved residues in the active site of insect dsRNases and bacterial NsNuc (Figure 4) suggests that there may be differences in substrate specificity among the dsRNases. Based on the crystal structure obtained for a bacterial nonspecific nuclease from *Serratia marcescens*, the first, second, and eighth key residues in the dsRNase active site are likely involved in substrate binding [38]. Variability at these residues may indicate differences among OndsRNases and DvdsRNases in substrate specificity and activity under physiological conditions, as described for LmdsRNases in *L. migratoria* [34,35]. 

OndsRNase1 and OndsRNase2 both have an alanine (A) and an arginine (R) at positions 1 and 2 in the active site similar to LmdsRNase3 (Figure 4), which was shown to actively degrade both dsRNA and dsDNA under physiologically relevant conditions in *L. migratoria* hemolymph [35]. Interestingly, BmdsRNase3 from *B. mori*, and TcdsRNase1 from *Tribolium castaneum* also have an alanine and arginine at positions 1 and 2 (Figure 4). BmdsRNase3 was first reported to have high activity against dsRNA and to a lesser extent siRNA under physiologically relevant conditions in *B. mori* gut contents [29], but later reported to have activity against dsRNA and DNA in lepidopteran HiF tissue culture cells [39]. These reports from *B. mori* offer further support that alanine and arginine at these positions in the dsRNase binding site is associated with dsRNA specificity of dsRNase enzymes. The substrate specificity of individual TcdsRNases from *T. castaneum* has not been investigated yet, but dsRNA and dsDNA have been reported to be relatively stable in *T. castaneum* gut contents and hemolymph [40]. Perhaps, the physiological pH in *T. castaneum* tissues is not suitable for dsRNase activity in this species, as was shown for LmdsRNase1 and 4 in hemolymph [35] and LmdsRNase3 in gut contents [34]. Based on the data presented in Figure 5 and this body of literature, it is reasonable to predict that OndsRNases2 and OndsRNase1 may have substrate specificity for dsRNA and dsDNA in ECB. 

Conversely, OndsRNase3, OndsRNase4, and DvdsRNase1 all have a serine (S) and arginine (R) at positions 1 and 2 in the active site, like LmdsRNase4 (Figure 5), which was shown to slightly degrade siRNA and dsRNA in vitro, but not at physiologically relevant conditions in hemolymph [35]. DsRNase1 from *B. mori* and TcdsRNase2 from *T. castaneum*, also have a serine and arginine at positions 1 and 2. The contribution of BmdsRNase1 to dsRNA instability in *B. mori* has not been investigated, but dsRNA is relatively stable in tissue extracts from *D. v. virgifera* [14] and *T. castaneum* [40]. Based on dsRNA stability data and the literature discussed here, it is reasonable to speculate that OndsRNases3 and OndsRNase4 may have a slight substrate specificity for dsRNA and siRNA, but likely are not contributing greatly to dsRNA instability in ECB. LmdsRNase2, the enzyme responsible for dsRNA degradation in gut contents and for lowering oral RNAi efficiency in *L. migratoria* [34], has a serine (S) and a lysine (K) at positions 1 and 2 of its active site, which may be unique to orthopterans (Figure 5) [35], but more investigation is necessary to be sure. 

These hypotheses regarding OndsRNase specificity and activity are further supported by the developmental stage (Figure 5) and tissue (Figure 6) specific expression profiles generated for dsRNase transcripts in ECB, which showed very high expression of *OndsRNase2* in the larval gut, very low expression of *OndsRNase3* and *OndsRNase4* in most stages and tissues, and high expression on *OndsRNase1* in all tissues, mainly in older developmental stages. Together these findings indicate *OndsRNase2* and *OndsRNase1* are the most likely dsRNase genes to contribute to low dsRNA stability in ECB. 

Given that *OndsRNase3* expression was nearly undetectable in all developmental stages and tissues investigated (data not shown), and the protein contains a highly abnormal lysine (K) at position 8 in the substrate-binding pocket (Figure 5), this nuclease may be nonfunctional (although lysine and arginine are both positively charged amino acids, so it is possible that the protein could be functional), and is unlikely to contribute to low RNAi efficiency in ECB; however, testing of activity and substrate specificity with heterogeneously expressed enzymes, or incubation of various substrates in extracts from transgenic ECB lacking specific dsRNases is needed to confirm these hypotheses. In addition, a variety of other obscure nuclease genes may contribute to dsRNA degradation, as shown in *Nezara viridula* [41] and *H. armigera* [14]. Information about nucleases will aid in the development of strategies for enhancing dsRNA stability in insect tissues, with the ultimate goal being to enhance RNAi efficiency and reduce the dose of costly dsRNA needed to induce phenotypical changes associated with suppression of the target gene.

The low transcript levels of dsRNase genes in early-instar larvae and adult females of ECB suggests that these developmental stages may be the most amenable to RNAi, as dsRNA is likely the most stable in these developmental stages [11]. However, this hypothesis relies on the assumption that dsRNase expression is indeed correlated with dsRNA stability in vivo. A comparison of RNAi efficiency and/or dsRNA stability in tissues from various developmental stages of ECB is needed to confirm this. Additionally, the elevated transcript levels of *OndsRNase4, OndsRNase1,* and *OnREase* in adult males is also noteworthy (Figure 5 and Figure 7A) and deserves investigation.

*OndsRNase2* was not upregulated at any of the time points investigated (data not shown). This result is surprising given the high basal expression in the larval gut (Figure 6A and Figure 7A) and leads to questions about the importance of gene inducible vs. constitutive expression. Lack of upregulation does not necessarily indicate that nucleases such as *OndsRNase2* are not involved in dsRNA degradation or defense against invading dsRNA. In *B. mori* larvae, *BmdsRNase* was upregulated 3 and 6 h after dsRNA injection, but not ingestion [31], despite high expression and activity of this nuclease in the midgut [29,30]. However, upregulation of genes such as *OndsRNase4* (Figure 8) and *OnREase* (Figure 10) in response to dsRNA injection may indicate that these enzymes act as pattern recognition receptors, similar to *BmdsRNase*, which was shown to mimic the response of Toll receptors and core RNAi pathway components to pathogen-associated molecular patterns such as dsRNA [31]. 

The transcription response of REases to exogenous dsRNA has been the subject of much interest because induction of this nuclease occurs sooner and higher than upregulation of the core enzymes in response to dsRNA in *O. furnicalis*, thus limiting the amount of dsRNA that reaches the core enzymes of the RNAi pathway and thus reducing RNAi efficiency [12]. While dramatic upregulation of *OfREase* and *HaREase* in response to orally delivered dsRNA is well documented [12,13], this is the first report of upregulation of a REase gene in response to injected dsRNA (Figure 10A,B). This finding may explain why *O. furnicalis* and *H. armigera* larvae are generally amenable to RNAi via injection and soaking [12,42], whereas ECB is much less susceptible (pending publication). 

The downregulation of *OndsRNase1* in response to the Zn^2+^ nuclease inhibitor (Figure 9) supports the existence of a feedback loop between perturbations in nuclease activity and expression of nuclease genes. In ECB, injection of a nuclease inhibitor resulted in downregulation of *OndsRNase1*, but in *H. armigera* knockout of *HaREase* resulted in upregulation of 14 other nuclease genes [13]. Therefore, protective coatings such as nanoparticles and transfection reagents may be better candidates for combating dsRNA-degradation and enhancing RNAi efficiency in insects because they are unlikely to impact nuclease activity or expression. 

Taken together, the results presented in this investigation are consistent with studies that characterize nuclease genes from other insects. There is a high probability that instability of dsRNA in ECB could be due to rapid degradation of dsRNA by at least some of the identified dsRNases and REase in the gut and hemolymph. Nuclease inhibitors [40,41,42,43], transfection reagents [44,45], dsRNA-guanylated polymer complexes [46], dsRNA-expressing bacteria [47], and ribonucleoprotein-dsRNA [48] can protect dsRNA from nuclease activity in insects such as *Blattella germanica*, *Euschistus heros, Anthonomus grandis, Acyrthosiphon pisum,* and *H. armigera*. These reagents and approaches are good candidates for enhancing dsRNA stability in ECB, and hopefully RNAi efficiency as well, so that RNAi-based approaches can be utilized to study and control ECB in the future. However, it might prove challenging to inhibit intracellular nucleases such as OnREase without affecting the function of core RNAi pathway genes, such as Dicer 2 or Argonature 2. There could also be additional mechanisms contributing to low RNAi efficiency in ECB [reviewed in 11] that will need to be understood and overcome before RNAi efficiency can be employed in control strategies against lepidopteran insects like ECB. 

## 5. Conclusions

This investigation identified transcripts for four dsRNase genes and one REase gene expressed in ECB. Comparison of key amino acid residues in the predicted active site of the nonspecific endonuclease domains of the dsRNases also suggested that OndsRNase1 and OndsRNase2 likely have substrate specificity for dsRNA and dsDNA, whereas OndsRNase3, and OndsRNase4, likely are specific to dsRNA and siRNA. In addition, expression profiles indicated that *OnREase*, *OndsRNase2* are most likely to contribute to dsRNA instability in the larval gut, whereas *OnREase* and *OndsRNase1* are most likely to contribute to dsRNA instability in the hemolymph. However, only *OnREase* and *OndsRNase4* were upregulated in response to dsRNA exposure, indicating that only a subset of these genes may act as pattern recognition receptors for dsRNA. Together, these findings support the idea that dsRNA degradation in ECB is likely due to the enzymatic activity of dsRNA-degrading nucleases in ECB. Further testing, however, is necessary to experimentally confirm the involvement of specific nuclease genes. These findings are significant because they provide information about the mechanism(s) influencing dsRNA instability in insects. This knowledge is useful for devising strategies to enhance dsRNA stability, and possibly RNAi efficiency in ECB. Thus, the knowledge generated by this study will facilitate the development of strategies for enhancing dsRNA efficiency insects.

## Figures and Tables

**Table 1:** Characteristics of five nuclease transcripts and their deduced proteins from European corn borer (ECB). The likelihood and/or cleavage site of the Sec signal peptide (Sec/SPI) based on the Signal-P-5.0 server is listed first, and then the likelihood/cleavage site based on the Target-P-2.0 server. The localization predictions with the highest probabilities according to WoLF PSORT, Euk-mLoc 2.0, and iLoc-Animal are listed.

Gene Names	*OndsRNase1*	*OndsRNase2*	*OndsRNase3*	*OndsRNase4*	*OnREase*
GenBank accession number	MT524715	MT524712	MT524713	MT524714	MT524716
ORF (bp)	1341	1347	1182	1212	1866
Protein (aa)	446	448	393	403	621
Mass (kDa)	50.66	50.01	44.13	46.14	71.99
Isoelectric point (pI)	5.67	7.17	7.95	6.26	5.64
Sec/SPI likelihood	0.9956, 0.999	0.9978, 1.000	0.9948, 0.997	0.7565, 0.9536	0.0013, 0.0005
Sec/SPI cleavage site (aa)	18–19	16–17	20–21	22–23, 23–24	n/a
Localization predictions	Extracellular, NASL	Extracellular, Mitochondria	Extracellular, NASL	Extracellular, NASL, Mitochondria	Cytoplasm, Nucleus

Abbreviations: dsRNase, double-stranded ribonuclease; ORF, open reading frame; bp, base pair; kDa, kilodalton; aa, amino acid; NASL, not in any subcellular locations; Sec/SPI, Sec signal peptide.

## Supplementary Materials

The following are available online at https://www.mdpi.com/2075-4450/11/10/652/s1, Table S1: Primers for dsRNA synthesis, cDNA synthesis, and RT-qPCR to investigate nucleases in ECB, Figure S1: Multiple sequence alignments showing conserved domains, residues, and signal peptides in insect dsRNase proteins, Figure S2: Multiple sequence alignments showing conserved domains and residues in insect REase proteins.
