# Supplementary material for: Molecular Characterizations of Double-Stranded RNA Degrading Nuclease Genes from Ostrinia nubilalis
Source: Insects. 2020 Sep 23;11(10):652. doi: 10.3390/insects11100652 (PMC7598268; doi:10.3390/insects11100652)
Supplement: Supplementary file 1 [file insects-11-00652-s001.pdf]

Table 1. Primers for dsRNA synthesis, cDNA synthesis, and RT-qPCR to investigate nucleases in ECB.

Application of primers	Target gene	GenBank accession no.	Sequence of primers (5′to 3′)	Product size (bp)	%E
cDNA cloning	*OndsRNase2*	MT524712	F: GCCCTGACTACACTGAAGGTG<br>R: ACGCCCCTATTACGGTGACTGATGG	1,454	
	OndsRNase3	MT524713	F: TAACCCAAGCAATACCTAT<br>R: TATGTAAACCAGGGATGT	1,281	
	OndsRNase4	MT524714	F: ATGATACATCTGCAAAAACTATTTACTC<br>R: TGTGCCTATTTAAGACCG	1,280	
	OndsRNase1	MT524715	F: ATGCACTCCGTCGTGGTGTTTC<br>R: GTAAGGGGTCTGCTGAACTAAG	1,342	
	OnREase	MT524716	F: CACAATGCGGATACGAGC<br>R: CTGCTTACTTACAAATTGGTC	1,878	
	DvdsRNase1	MT653318	F: ATGGTAAGATATAGGTGTG<br>R: CTAAGCTTGTAAAATACC	1,209	
	DvdsRNase2	MT653319	F: GGTATTATCGCGGTCTACT<br>R: TGTTATTCATAAATAGCCCG	1,628	
	DvdsRNase3	MT653320	F: GATGCGATCCATGCATTGT<br>R: GGTCGCAACTATGACTCTTT	1,417	
dsRNA synthesis	*OnLgl*	MT467568	F: taatacgactcactatagggagCCAACCAGCAGTTGGAGAGT<br>R: taatacgactcactatagggagAGGTAAGGCAACCTCATTGG	506	
	AvGFP	LC336974.1	F: taatacgactcactatagggagCTAGAGTGAGCAAGGGCGAG<br>R: taatacgactcactatagggagCTTGAAGTTCACCTTGATGCC	503	
RT-qPCR	*OndsRNase2*	MT524712	F: AGCAGCCAAGAGTGACTTCC<br>R: GGGCACGGAGATTCTGTTCA	128	97.8
	OndsRNase3	MT524713	F: ACGGTTGCGGAAATCGGAA<br>R: CCCACTCGGAAAGTGGTGT	132	95.4
	OndsRNase4	MT524714	F: TCGTTGTTTTCACCGCGAG<br>R: GAAGTCTGTCTTCGCCGCA	123	99.4
	OndsRNase1	MT524715	F: TGGCGATTTCTACCCTGGC<br>R: CGCGTGCCAAGTACTGACT	131	104.1
	OnREase	MT524716	F: ACTCCAGGCGAGCCTGTCAA<br>R: GCATCGTCAGCGTCCGGATCCTC	182	n/a
	OnRPS3	DQ988989	F: CTGGCCGAAGATGGTTACTC<br>R: ACCACGGAGGTTAGTTCACG	134	89.2

OnEF1a	AF173392	F: CCCGCTAACCTGACCACT<br>R: AAACCACGACGCAACTCC	128	95.3

Abbreviations: bp, base pair; cDNA, complementary DNA; dsRNA, double-stranded RNA; RT-qPCR, reverse transcription quantitative real-time PCR; ORF, open reading frame; *On, Ostrinia nubilalis; Dv, Diabrotica virgifiera virgifera,; Av, Aequorea victoria; Lgl, lethal giant larvae; GFP, enhanced green fluorescent protein*; ECB, European corn borer; WCR, western corn rootworm, %E, percent primer efficiency; F, forward primer; R, reverse primer; RPS3, *Ribosomal protein S3; EF1a, Elongation factor 1-alpha.*

```
OndsRNase1 : MH-SVVVF-LAVVFATAVKAADHLD--DPSDLALTLEEEEFENFLDEYLTIEELSW----ISEPEDDEEETVCIFKIRGDLGQPQPVYVHNNKLLEPAGNTGQVRVTAGQEIIIA : 107
OndsRNase2 : MR-TLVIFACAVLAVTAMPA----DLPEPGELALMLNEEXFEDYLDEWLAKEEPNWVNLTLADEQRNG-RSGCTFRVNGDLGQPQPVYIRRNQLLMPSGNTGQIFVNTGEQIRIA : 109
OndsRNase3 : -----------------------------------------------MKCVSMVFCFTALLLAISIADVRGECKLSLQDDFKKPAPVYIKDGVFLAPNPA-GDIRLRKSETLAVA :  67
OndsRNase4 : -----------------------------------------------MIHLQKLFTLILCVFNIQPGQR---CRLHSRTHFGEPLPVIIRNGKLLEPTDNFGNVDMEYGDSMTLS :  65
BmdsRNase1 : -------------------------------------------------MILKYLMMILCVFNVKPGQR---CILNSRRDFGQPLPVIIRNGRLLEPTDKYGNVEIDNGETLTLS :  63
BmdsRNase2 : ---------------------------------------------------------------------------------------------------------------MVVA :   4
BmdsRNase3 : MRLTLVLAALAV-AVVALPSKLRTDLPEPGQLAFVLNEDEFEDYLDAYLALQQSE----MLANQTRNDFRSGCTFRVNGDLGQPQPVYIHRGNYLSPTGNTGQIRLNRGEQVLIA : 110
TcdsRNase1 : ------------------------------------------------------------------------------------------------------------------- :   -
TcdsRNase2 : ------------------------------------------------------------------------------------------------------------------- :   -
DvdsRNase1 : ------------------------------------------------------------------------------------------------------------------- :   -
DvdsRNase2 : ------------------------------------------------------------------------------------------------------------------- :   -
DvdsRNase3 : ------------------------------------------------------------------------------------------------------------------- :   -
LmdsRNase1 : ------------------------------------------------------------------------------------------------------------------- :   -
LmdsRNase2 : ------------------------------------------------------------------------------------------------------------------- :   -
LmdsRNase3 : ------------------------------------------------------------------------------------------------------------------- :   -
LmdsRNase4 : ------------------------------------------------------------------------------------------------------------------- :   -

OndsRNase1 : CPGQNNKIRHPKIISD---VAFAKATCMNGTIISGVGWLRDEGEFGRLTCARHPESQAVLTNEECFDHNTVI------------------------------------------- : 176
OndsRNase2 : CTGSGRTIQHPNINAN---VAVATATCVSGNTVSGSGWLRANGAFGGLTCSAHAFHEAQNTNDRCFNNHAVI------------------------------------------- : 178
OndsRNase3 : CPGNKRRVVLGNVTTS---LDVVEASCVSNTTFRVGQWL---GPFKSITCNVQPWFSSQEVKGGCGRGNQLH------------------------------------------- : 133
OndsRNase4 : CEGTGY-ILHPDTKTN---VITASVSCLGGDNFGNDQWLNGPARFTMFKCLYPPNHLSQRTNRTCFEGNPVI------------------------------------------- : 133
BmdsRNase1 : C-GESR-VRHPNANKH---FEVATVTCQGGDTFTNNDWITAPSSFLFFSCDIPPVYMSKRTNRTCHNNNKIF------------------------------------------- : 130
BmdsRNase2 : CPGSRNHIVLGNETGKGRQFDVLDAKCVEKTSFLLGGWR---GEFRNVTCKTQPWTTVYDTKKICHLNSKLY------------------------------------------- :  73
BmdsRNase3 : CTGSGRTIRHPNVASN---LAVGTVSCQNNNLVT-ANWLRGNSAFGQLTCSSHAYHDAQQTNTRCFNNHFVI------------------------------------------- : 178
TcdsRNase1 : ----------------------------------------------------------------------------------------------------------MLLPALFLL :   9
TcdsRNase2 : ----------------------------------------------------------------------------------------------MANLYSYK--MKLFLVG--CV :  17
DvdsRNase1 : -------------------------------------------------------------------------------------------------MVRYRCVFSWLILG-ACS :  17
DvdsRNase2 : ----------------------------------------------------------------------MIYFSKPFLKSRKFFLIVTGVVVGIVLLIIIIVAASETHTGTKSS :  45
DvdsRNase3 : -------------------------------------------------------------------------------------------------MRSMHCFLSATMNSVIII :  18
LmdsRNase1 : ------------------------------------------------------------------------------------------------------------------- :   -
LmdsRNase2 : -------------------------------------------------------------------------------------------------------MASLPLPLVLAA :  12
LmdsRNase3 : -------------------------------------------------------------------------------------MWTRGRVVQVCALVVVAAVANLQ-PGDAAA :  29
LmdsRNase4 : ------------------------------------------------------------------------------------------------------------MERTAAS :   7

OndsRNase1 : ------------------------------------------------------------------------------------------------------------------- :   -
OndsRNase2 : ------------------------------------------------------------------------------------------------------------------- :   -
OndsRNase3 : ------------------------------------------------------------------------------------------------------------------- :   -
OndsRNase4 : ------------------------------------------------------------------------------------------------------------------- :   -
BmdsRNase1 : ------------------------------------------------------------------------------------------------------------------- :   -
BmdsRNase2 : ------------------------------------------------------------------------------------------------------------------- :   -
BmdsRNase3 : ------------------------------------------------------------------------------------------------------------------- :   -
TcdsRNase1 : LQVSLGDFFILRAPDCNIQ--ISNL-----DPEPIVVD-GTYTFLYA--APDASS--VLVKSGETIIISCPGGEITVGSTSFNSTVSATCVSNSDFSVGSATINFNQIVCSWNPF : 112
TcdsRNase2 : FIAYFVHVLVANQKGCSFS--VHGQS-E--KNSPILLQNDTT-----LIVPNN--GKVSLKRRDTVTLLCPDSKNYLVGTQ-SNITQAQCVQGGILRVANQDLTFRDLQCKKIIR : 119
DvdsRNase1 : QFGY-----SASRAGCTFN--VYNNANE--KYMPVMLTNHSSK--YELIVPEQ--GQIHLRSGEGITFVCPE-KNYLVLTN-SNFTYATCIQGTNLKLFRNTYNFDSFLCSKSIR : 117
DvdsRNase2 : NVEDHTDEPNEPSGPCEINQI------QYRNTHPLLLSPKTDN----VIYPQGKANTI-FASNEEVEFSCSQSEVRANGVNLGSNITVVCKSNETFTYNGTNIEWKNVACSKTLV : 149
DvdsRNase3 : LVGYLLSVGTCATTGCEIN--------PFGSKAPLVVKYGTSS----LIEPTPGYTTLSFTRNAAVEFACPGTQIIKNGTSLGDIVTATCKTGDVFNINGHAVHWSYLSCNNVIN : 121
LmdsRNase1 : ----------------------------MPSPQPVLLSSGYAQDFSSYLLPNR-KGNLTVRRGQRLTVACPGSSFRI-LSGSRDHVAADCVGDTVFSVDSVDYTLNGLVCSSAAT :  85
LmdsRNase2 : LLATAGHASPLPRAGCSVD--VSG-S-DMPDPQPLLLKPGGSKDVHGFVTPDS-SGVISLDQNEQIIVACPD--NSLQVTG-QSQATATCVSGTTFSIDGQSYDFGDLRCRSQPK : 119
LmdsRNase3 : LLATPGRAG-----GCSVD--INDDS-SFPDPQPLFLLPGGSGGAKAFWQPDATSGLLTLEAGEQLLLACPGRGNLLTALD-VQEATATCVSGSTFSVGDQQFALQDLSCSHLPT : 135
LmdsRNase4 : QIIFLASFVILVHRGCSADCSVNVNSGDFPSPQPLLLRVGSDRDFAAFVTPDS-SGNISVSAGTKLLLACPN--ATIALLD-AESAEATCVSGSVFSVAGTPYTLNVLACSQLLK : 118
```

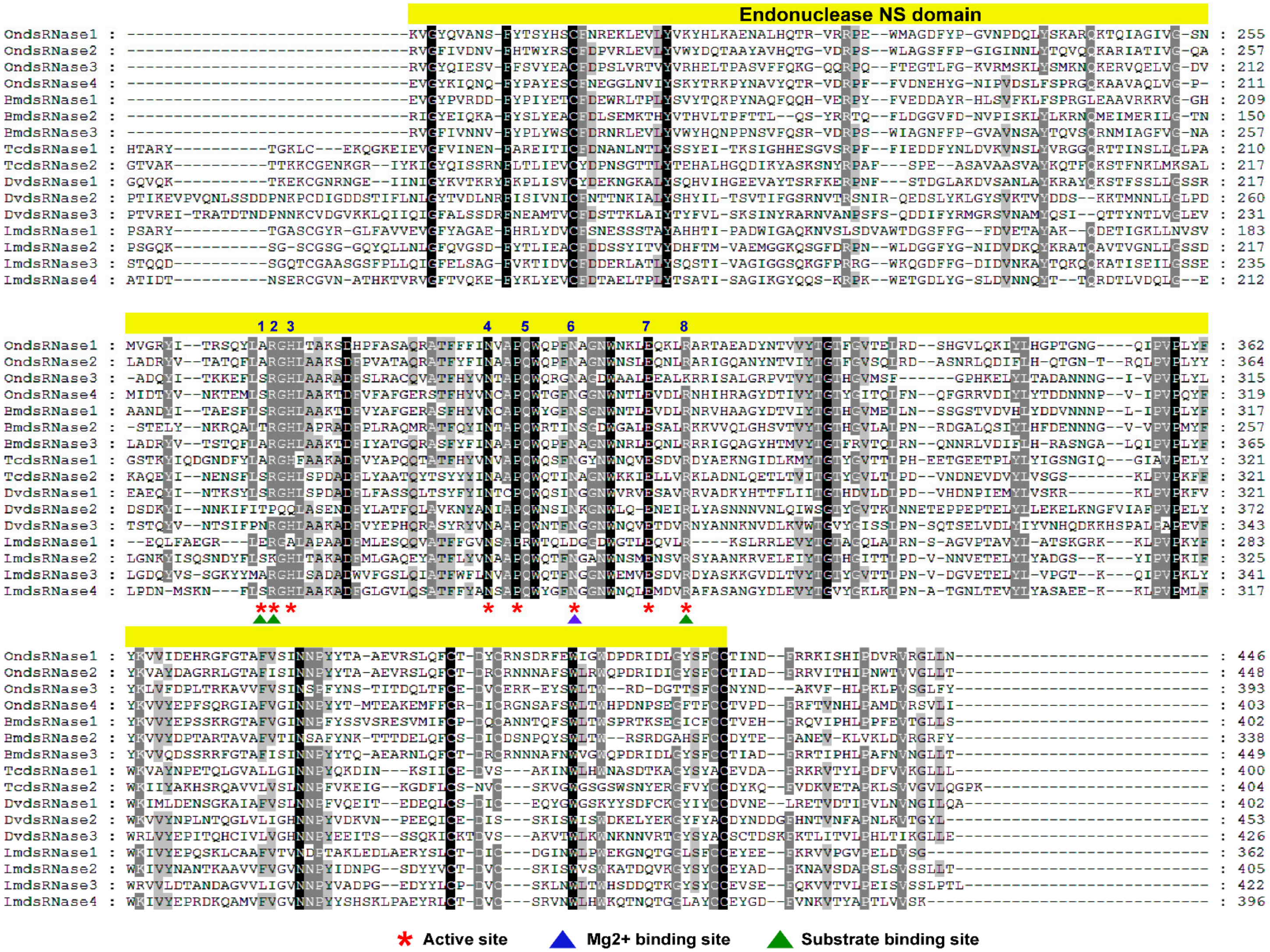
Endonuclease NS domain
OndsRNase1
OndsRNase2
OndsRNase3
OndsRNase4
BmdsRNase1
BmdsRNase2
BmdsRNase3
TcdsRNase1
TcdsRNase2
DvdsRNase1
DvdsRNase2
DvdsRNase3
ImdsRNase1
ImdsRNase2
ImdsRNase3
ImdsRNase4
1 2 3 4 5 6 7 8
Active site
Mg2+ binding site
Substrate binding site

Figure 1. Multiple sequence alignments showing conserved domains, residues, and signal peptides in insect dsRNase proteins. Extracellular secretion (Sec/SPI signal) peptides are indicated in red font and the position of the DNA/RNA non-specific endonuclease (endonuclease NS) domain by a yellow bar. The eight amino acid residues that form the active site are indicated by a red asterisk and numbered along the top. Amino acid residues that participate in the substrate-binding site and Mg^{2+} binding site are indicated with green and blue triangles, respectively. Black shading indicates 100% identity, dark-grey shading indicates 80–99% identity and light-grey shading indicates 60–79% identity. The species and gene accession number corresponding to each sequence label is as follows: OndsRNase1, *O. nubilalis* (MT524715); OndsRNase2, *Ostrinia nubilalis* (MT524712); OndsRNase3, *O. nubilalis* (MT524713); OndsRNase4, *O. nubilalis* (MT524714); BmdsRNase1, *B. mori* (XP_004922835.1); BmdsRNase2, *Bombyx mori* (NP_001091744.1); BmdsRNase3/AlkNuc, *B. mori* (BAF33251.1); TcdsRNase1, *Tribolium castaneum* (XP_970494.1); TcdsRNase2, *T. castaneum* (XP_015840884.1); DvdsRNase1, *Diabrotica virgifera virgifera* (MT653318); DvdsRNase2, *D. v. virgifera* (MT653319); DvdsRNase3, *D. v. virgifera* (MT653320); LmdsRNase1, *L. migratoria* (ARW74134.1); LmdsRNase2, *L. migratoria* (ARW74135.1); LmdsRNase3, *Locusta migratoria* (KY386893); LmdsRNase4, *L. migratoria* (KY386894).

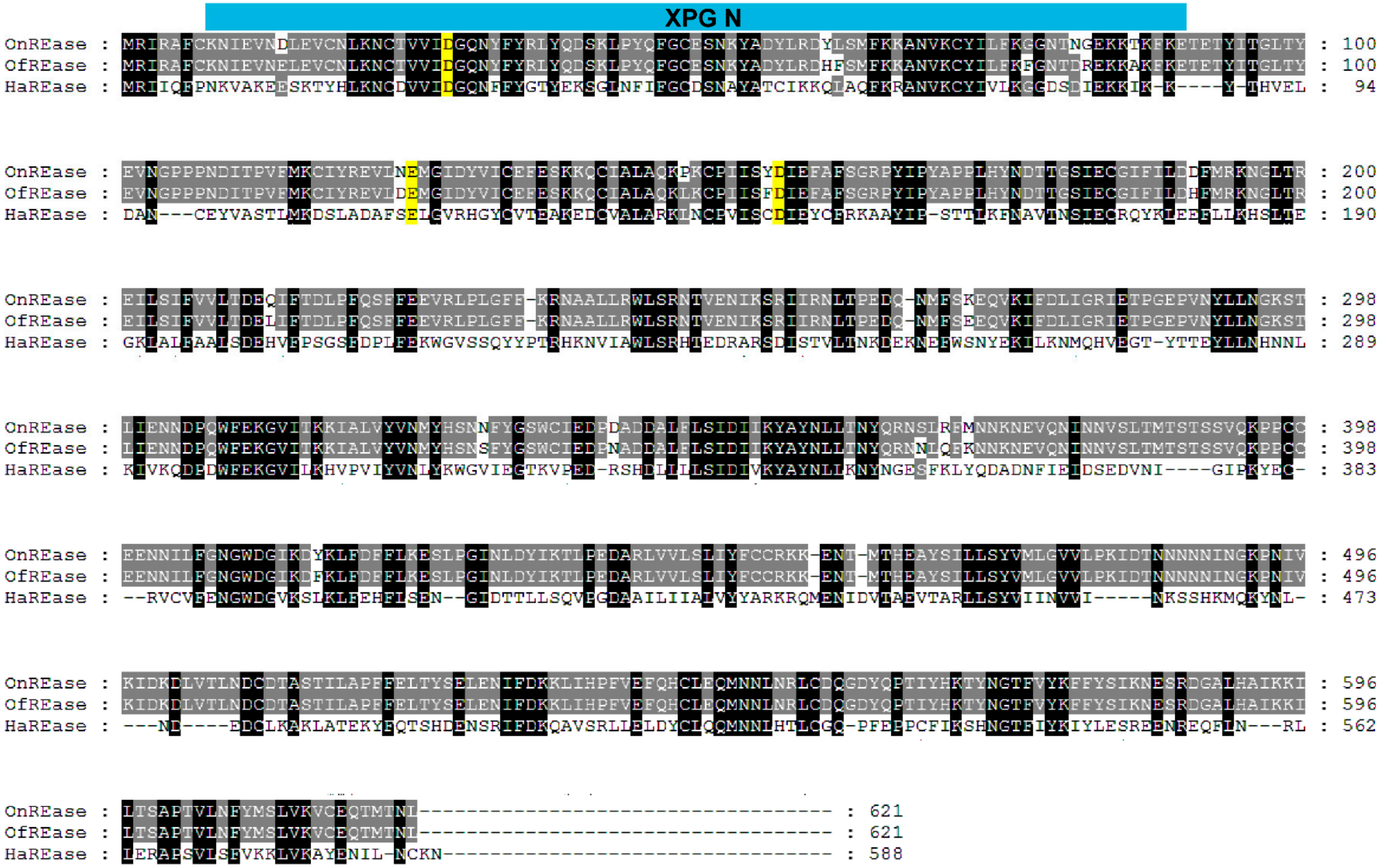

Figure 2. Multiple sequence alignments showing conserved domains and residues in insect REase proteins. The position of the XPG N-terminal domain is indicated by a blue bar and conserved PIN-domain family residues are highlighted. Black shading indicates 100% identity and dark-grey shading indicates 66% identity. The species and gene accession numbers corresponding to each sequence label is as follows: OnREase, *Ostrinia nubialis* (MT524716); OfREase *Ostrinia furnacalis* (XP_028162616.1); HaREase, *Helicoverpa armigera* (XP_021192733.1).
